# An Efficient VLSI Architecture for Multi-Channel Spike Sorting Using a Generalized Hebbian Algorithm

**DOI:** 10.3390/s150819830

**Published:** 2015-08-13

**Authors:** Ying-Lun Chen, Wen-Jyi Hwang, Chi-En Ke

**Affiliations:** Department of Computer Science and Information Engineering, National Taiwan Normal University, Taipei 116, Taiwan; E-Mails: eternity79831.unknown@gmail.com (Y.-L.C.); 60147074s@ntnu.edu.tw (C.-E.K.)

**Keywords:** spike sorting, VLSI, brain machine interface

## Abstract

A novel VLSI architecture for multi-channel online spike sorting is presented in this paper. In the architecture, the spike detection is based on nonlinear energy operator (NEO), and the feature extraction is carried out by the generalized Hebbian algorithm (GHA). To lower the power consumption and area costs of the circuits, all of the channels share the same core for spike detection and feature extraction operations. Each channel has dedicated buffers for storing the detected spikes and the principal components of that channel. The proposed circuit also contains a clock gating system supplying the clock to only the buffers of channels currently using the computation core to further reduce the power consumption. The architecture has been implemented by an application-specific integrated circuit (ASIC) with 90-nm technology. Comparisons to the existing works show that the proposed architecture has lower power consumption and hardware area costs for real-time multi-channel spike detection and feature extraction.

## 1. Introduction

Neurons are the basic elements that underlie the function of the nervous system, which contains the brain, spinal cord and peripheral ganglia. Neurons process and transmit information mainly by electrical signaling through the generation of action potentials. These action potentials can be recorded *in vivo* by placing electrodes in the vicinity of the neurons. The spikes recorded by the electrodes represent spike events generated by an unknown number of neurons. The role of spike sorting [1,2] is to assign each spike to the neuron that produced it. A typical automatic spike sorting involves complicated operations, such as spike detection, feature extraction and classification. As the technology progresses, multi-channel arrays are increasingly being employed. Increasing the number of recording electrodes raises the computation time for automatic spike sorting, as detection and feature extraction become tedious tasks. However, for many spike sorting applications [3], real-time operations are desired. Hardware systems offering dedicated circuits can substantially outperform their software counterparts in terms of computational performance and power dissipation. Hardware solutions are therefore necessary for neurophysiological signal recordings and analysis, where these factors are crucial.

One effective technique for spike sorting is based on principal component analysis (PCA) [4,5] for feature extraction. Implementation of PCA-based hardware systems [6,7] involves the computations for covariance matrix and eigenvalue decomposition. Therefore, direct realization of PCA requires substantial hardware costs, such as power consumption and hardware area. Alternatives to PCA, such as discrete derivatives [8], integral transform [9] and zero-cross features [10], have been proposed to reduce the computational complexities for feature extraction. However, their effectiveness remains to be validated through real data experiments.

Besides feature extraction, spike sorting requires a set of prepossessing steps, including spike detection and spike alignment. Spike detection distinguishes neuronal spikes from background noises. A commonly-used detection method is to compare the absolute voltage of the recorded signal with a threshold [11,12]. However, the method may not performs well for spike trains corrupted by large noises. The nonlinear energy operator (NEO)-based [13] spike detection method detects the peak of a spike by simple arithmetic operations. It provides a hardware-efficient alternative and also achieves high detection accuracy by considering both spike amplitude and frequency. Spike alignment can be carried out by the positions of the peak or maximum slope of the detected spikes [1]. Because the peak location of the detected spikes can be obtained from the NEO, the peak-based spike alignment operating in conjunction with the NEO may be more hardware friendly as compared to its counterparts using maximum slope techniques.

The objective of this paper is to present a novel VLSI architecture capable of performing *in vivo* multi-channel spike sorting. In the architecture, the spike detection and alignment are based on NEO with peak-based alignment, and the feature extraction is carried out by the generalized Hebbian algorithm (GHA) [14]. The GHA can be viewed as an incremental PCA, which computes the principal components without the involvement of the covariance matrix. In the GHA, the principal components are updated incrementally based on a set of training data. For the spike sorting applications, the training set is formed by the detected spikes. In the hardware implementation for GHA, only the buffers storing the principal components and the arithmetic operation circuits for updating principal components are required. No memory for storing the covariance matrix of the training set is necessary. Power consumption and hardware area can then be reduced substantially, as compared to its counterparts based on the batch PCA algorithm.

Although a number of GHA hardware architectures [15,16] have been proposed, the circuits are implemented for single channel spike sorting only. One simple way to extend the circuits to multi-channel cases is to replicate the circuits, one for each channel. Designs in this way may result in high power dissipation and large hardware area. In addition, the existing implementations are by field programmable gate array (FPGA) [17], which may not be well suited for the realization of bio-implantable spike sorting systems due to the large power consumption inherited from FPGAs.

The proposed NEO and GHA circuits are implemented by an application-specific integrated circuit (ASIC) [18] to lower the power consumption. In addition, they are designed specifically for multi-channel spike sorting. To minimize the power consumption and area costs of the circuits, all of the channels share the same core for spike detection and feature extraction operations. Each channel has dedicated buffers for storing the detected spikes and the principal components of that channel. A clock gating (CG) technique [19,20] is employed to supply the system clock only to the buffers of the channels currently using the computation core. The dynamic power dissipation of the inactive channels can then be further reduced. Furthermore, the relations among the sampling rate of spikes, the number of channels of the recording system and the latency of the GHA circuit are investigated in this study. A general guideline for optimizing the design is then derived. A number of design examples are provided to demonstrate the effectiveness of the proposed architecture. Experimental results reveal that the proposed architecture is an effective alternative for “*in vivo*” multi-channel spike sorting with low power dissipation and low hardware area costs.

## 2. The Architecture

### 2.1. Overview

**Figure 1:**
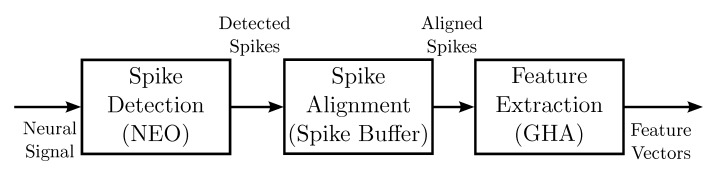
The general block diagram of the proposed architecture for spike sorting.

Figure 1 depicts the general flow of the proposed architecture for spike sorting. There are three operations supported by the proposed architecture: spike detection, spike alignment and feature extraction. The proposed architecture accepts raw spike sequences from different channels as input data. The goal of spike detection is to identify spikes from sequences using the proposed NEO circuit. The spike alignment operations first align spikes based on the location of their peak samples. The aligned spikes from different channels are stored in different buffers for subsequent feature extraction operations. For each detected spike, the proposed GHA circuit is used for extracting the feature vectors. The hardware circuits for detection, alignment and feature extraction are discussed separately in the following subsections.

### 2.2. NEO Circuit for Spike Detection

Let $s_{k}$ be the *k*-th sample of the input spike train of the single channel. In the NEO algorithm, a spike is detected from the channel when:

(1) $$s_{k}^{2}-s_{k-1}s_{k+1}>\gamma$$

where γ>0 is a threshold for spike detection. Without loss of generality, for some k>0, suppose that Equation (1) holds, and the corresponding spike is the detected spike, denoted by *x*, of the channel for GHA operations. Let $x_{i},i=1,...,m,$ be the *i*-th sample of **x**, where *m* is the dimension of the spike. Assume that $x_{P}$ is the sample in **x** having the peak value. Since $s_{k}$ satisfying Equation (1) is the $x_{P}$, it then follows that $x_{i}=s_{k-P+i},i=1,...,m$. Each detected spike will be used by GHA for feature extraction.

**Figure 2:**
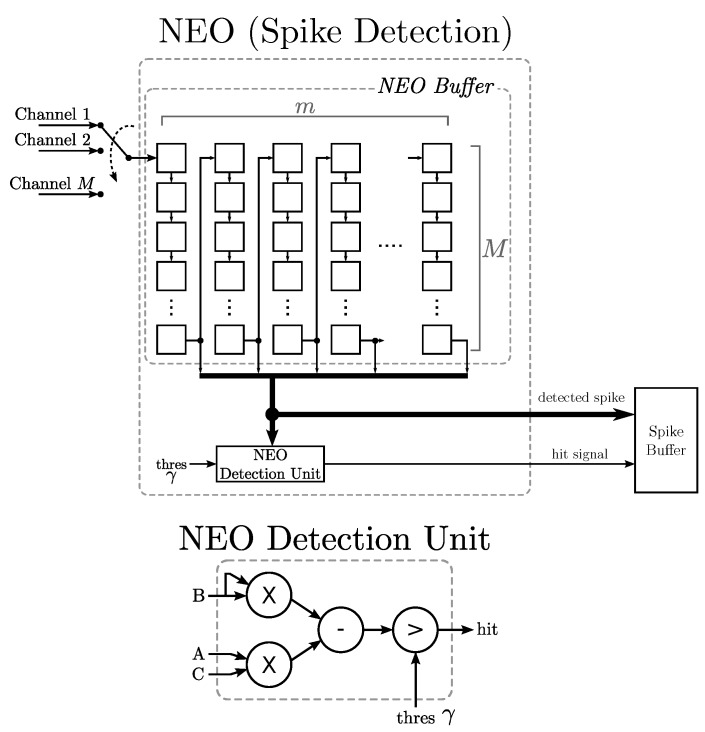
The architecture of the nonlinear energy operator (NEO) circuit for spike detection. The architecture contains the NEO buffer and the NEO detection unit. The NEO buffer is able to hold spike sequences up to *M* channels. All of the channels share the NEO detection unit.

From Equation (1), it can be observed that the NEO operations require only multipliers and adders for a single channel. The extension of the NEO circuit for a single channel to multiple channels can be carried out by simply replicating the circuit one for each channel. An alternative is to allow all of the channels to share the same circuit for NEO operations, as shown in Figure 2. In this way, the average area cost per channel may be reduced.

Let *M* be the number of channels for spike sorting. Assume all of the channels are sampled with the same sampling rate $r_{s}$. Let $T_{s}=1/r_{s}$ be the sampling period. All of the channels are assumed to be sampled and multiplexed by a mixed mode circuit using the round robin approach. The mixed-mode circuit then delivers the samples one at a time to the NEO circuit. Therefore, the NEO circuit receives *M* samples during a time interval of length $T_{s}$. Different samples received during the interval are from different channels.

The proposed NEO circuit can be separated into two portions: the NEO buffer and the NEO detection unit. The NEO buffer is a (M×m)-stage first-in-first-out (FIFO) shift register organized in a snake-like fashion. Each stage contains a sample of a spike train from a channel. It is therefore able to hold *m* consecutive samples of the spike trains from the *M* channels.

The bottom row of the buffer provides *m* consecutive samples of the spike train from a channel (say, channel *h*). It can be seen from Figure 2 that the bottom row of the NEO buffer is used for both NEO detection and peak alignment. The NEO detection unit takes three consecutive samples of the bottom row to carry out the computation given in Equation (1). The computation result is then compared to a given threshold *γ*. If the result is larger than the threshold, a hit event is issued. In addition, the entire last row is regarded as a detected spike (denoted by $x_{h}$) and is copied to the spike buffer for spike alignment.

The proposed NEO circuit is able to perform spike detection one channel at a time. After the spike detection of the channel *h* is completed in the current clock cycle, all of the spike samples already in the NEO buffer are shifted to the next stage, and a new sample from the next channel (selected in round robin fashion) enters the first stage of the NEO buffer. Therefore, in the next clock cycle, the last row of the buffer contains *m* consecutive samples of channel $\bar{h}$, where $\bar{h}=\left(h+1\right)\;\mathrm{mod}\;M$. This allows the spike detection for channel $\bar{h}$ to be carried out in the next clock cycle.

**Figure 3:**
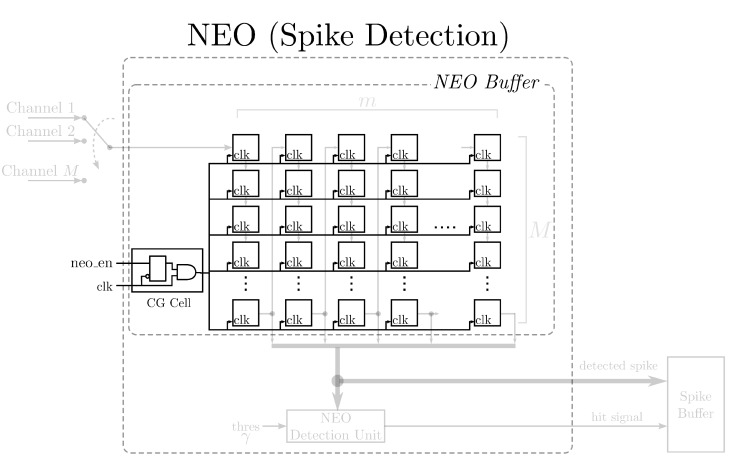
The clock gating circuit for lowering the dynamic power consumption of the NEO circuit for spike detection.

The power consumption of the proposed NEO circuit can be further lowered by the employment of the CG technique. This is because not all of the components of the proposed circuit need to be activated in each clock cycle. The CG technique operates by cutting off the system clock to the components that could be de-activated. The dynamic power consumption could then be reduced. The CG circuit employs the latches and AND gates for controlling the supply of the system clock. Figure 3 shows the augmentation of the CG circuit to the NEO circuit. As shown in Figure 3, the CG circuit controls the supply of the system clock to the NEO buffer of the NEO circuit. In a sampling period of $T_{s}$ seconds, the CG circuit turns off the system clock supply after all of the channels have fetched their new samples in the interval and restores the system clock at the beginning of the next sampling interval. The dynamic power consumption can then be effectively reduced.

### 2.3. Spike Buffer for Spike Alignment

**Figure 4:**
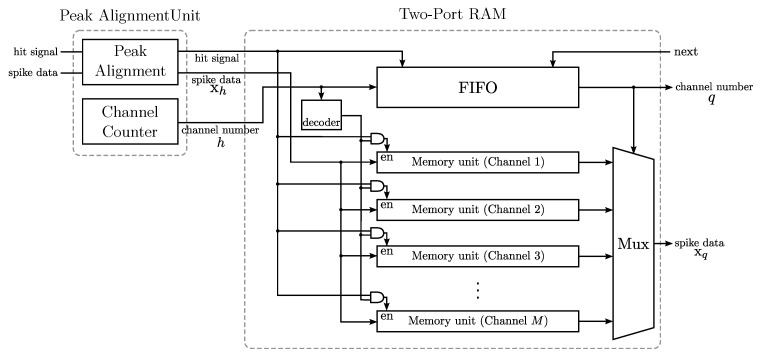
The architecture of the spike buffer for spike alignment. The architecture contains a peak alignment unit and a two-port RAM. The spikes aligned by the peak alignment unit are stored in the two-port RAM. The spike buffer is able to align and store detected spikes up to *M* channels.

The goal of the spike buffer is to hold the detected spikes produced by the NEO circuit, carry out the alignment and deliver the detected spikes to the GHA circuit upon the requests. Figure 4 shows the architecture of the spike buffer, which contains a peak alignment unit and a two-port RAM. The peak alignment unit is responsible for the spike alignment for each channel and the avoidance of multiple detection hits for a single spike. There are *M* memory units in the RAM, where each unit stores the detected spikes for one channel. The detected spikes from different channels are stored in different memory units. Each memory unit holds a single spike. That is, each memory unit consists of *m* registers, where each register contains the value of one spike sample. Moreover, each memory unit is a first-in first-out (FIFO) buffer for fast data access. In addition to the *M* memory units, the spike buffer comprises an FIFO buffer, which records the indices of the most recent channels issuing hit signals. The FIFO buffer is beneficial for providing the most recent detected spikes to the GHA circuit.

As shown in Figure 4, a hit event issued from the NEO circuit activates the spike buffer for a writing operation. The channel number *h* received from the NEO circuit serves as the index of the memory unit to which the detected spike is stored. The channel number *h* is also recorded in the FIFO buffer. A reading operation is initiated by the GHA circuit. Upon receiving a read request, the FIFO buffer provides the index of the channel, denoted by *q*, for the reading operation. The output of the spike buffer, denoted by $x_{q}$, is then delivered to the GHA circuit.

**Figure 5:**
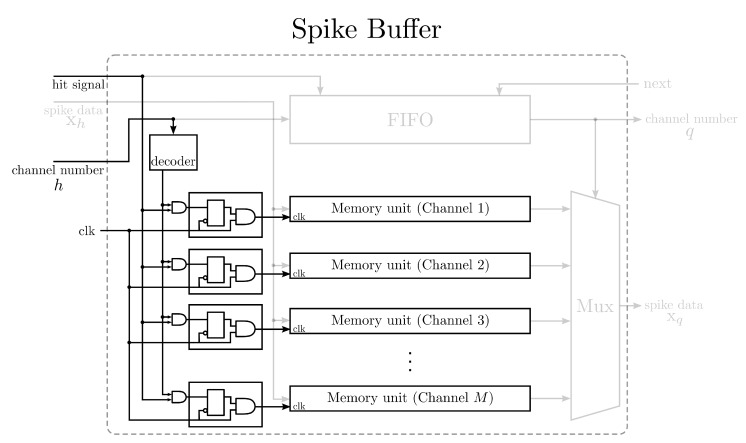
The clock gating circuit for lowering the dynamic power consumption of the spike buffer for alignment.

The CG technique can also be employed for reducing the power consumption of the spike buffer. Figure 5 shows the CG circuit for the spike buffer. It can be observed from Figure 5 that the CG circuit controls the supply of the system clock to each memory unit of the spike buffer. At most, one memory unit is operative at a time in the buffer. Therefore, each memory unit is kept inactive until it becomes the target of a writing operation initiated by the NEO circuit.

### 2.4. GHA Circuit for Feature Extraction

The GHA algorithm operates in two modes: training mode and service mode. The goal of training mode is to train the synaptic weight vectors $w_{j},j=1,...,p,$ for feature extraction, where *p* is the number of principal components. The $w_{j},j=1,...,p,$ and the detected spikes **x** have the same dimension *m*. After the training process is completed, the GHA can operate in service mode. Given a detected spike **x**, the GHA computes the feature vector $y={\left[y_{1},\ldots ,y_{p}\right]}^{T}$ in service mode, where $y_{j}$ is the inner product of $w_{j}$ and **x**. The subsequent clustering/classification operations are then based on the feature vector **y**.

Although the GHA in service mode is based on simple inner product operations, the GHA in training mode may require complicated operations for the updating of synaptic weight vectors. Let x(n), y(n) and $w_{j}\left(n\right)$ be the input training vector, the feature vector and the synaptic weight vectors at the iteration *n* of the training process, respectively. They are related by $y_{j}\left(n\right)=x^{T}\left(n\right)w_{j}\left(n\right)$, where $y_{j}\left(n\right)$ is the *j*-th element of y(n). Each synaptic weight vector, $w_{j}\left(n\right)$, is then adapted by the Hebbian learning rule:

(2) $$w_{ji}\left(n+1\right)=w_{ji}\left(n\right)+\eta \left[y_{j}\left(n\right)x_{i}\left(n\right)-y_{j}\left(n\right)\sum_{k=1}^{j}w_{ki}\left(n\right)y_{k}\left(n\right)\right]$$

where *η* denotes the learning rate and $x_{i}\left(n\right)$ and $w_{ji}\left(n\right)$ are the *i*-th element of x(n) and $w_{j}\left(n\right)$, respectively. After a great deal of iterative computation and adaptation, $w_{j}\left(n\right)$ will asymptotically approach the eigenvector associated with the *j*-th eigenvalue, $\lambda_{j}$, of the covariance matrix of input vectors, where $\lambda_{1}>\lambda_{2}>\cdots >\lambda_{p}$. To reduce the complexity of the computing implementation, Equation (2) can be rewritten as:

(3) $$w_{ji}\left(n+1\right)=w_{ji}\left(n\right)+\eta y_{j}\left(n\right)\left[x_{i}\left(n\right)-\sum_{k=1}^{j}w_{ki}\left(n\right)y_{k}\left(n\right)\right]$$

A more detailed discussion of the GHA algorithm can be found in [14]. In this subsection, the circuit for GHA in the training mode is presented. The circuit is also able to support GHA in service mode by deactivating its modules for synaptic weight updating.

**Figure 6:**
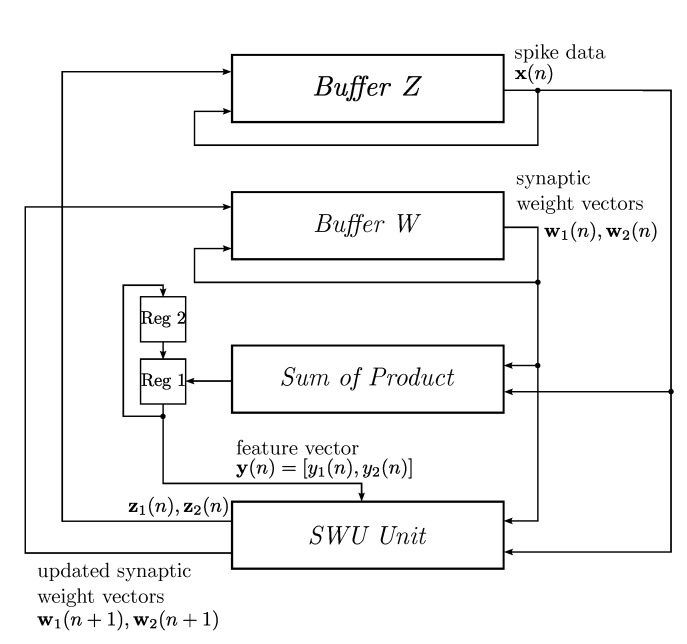
The architecture of the basic single-channel generalized Hebbian algorithm (GHA) circuit for feature extraction for p=2. The GHA circuit contains buffers, the sum of products (SOP) circuit and the synaptic weight vector updating (SWU) unit. The buffers store spike data and synaptic weight vectors. The SOP circuit computes feature vectors, and the SWU unit updates synaptic weight vectors. The SWU unit can be further separated into a number of modules.

The single channel case [16] is first considered. The proposed GHA circuit can be mainly separated into three portions: buffers, the sum of products (SOP) circuit and the synaptic weight vector updating (SWU) unit, as depicted in Figure 6. There are two buffers in the GHA circuit: Buffer W and Buffer Z. The synaptic weight vectors $w_{j}\left(n\right),j=1,...,p,$ and input spikes x(n) are stored in Buffer W and Buffer Z, respectively. Given the current synaptic weight vectors and the current input spike, the goal of the SOP circuit is to compute the feature vector $y\left(n\right)={\left[y_{1}\left(n\right),\ldots ,y_{p}\left(n\right)\right]}^{T}$, where $y_{j}\left(n\right)=x^{T}\left(n\right)w_{j}\left(n\right)$. This can be accomplished by an architecture consisting of *m* multipliers and an adder summing the *m* products produced by the multipliers.

After the operations of the SOP circuit are completed, the SWU is activated. Based on the feature vector y(n), current synaptic weight vectors $w_{j}\left(n\right),j=1,...,p,$ and the current input spike x(n), the objective of the SWU unit is to compute new synaptic weight vectors $w_{j}\left(n+1\right),j=1,...,p,$ using Equation (3). One way to implement Equation (3) in hardware is based on the observation that the equation can be rewritten as:

(4) $$w_{ji}\left(n+1\right)=w_{ji}\left(n\right)+\eta y_{j}\left(n\right)z_{ji}\left(n\right)$$

where:

(5) $$z_{ji}\left(n\right)=x_{i}\left(n\right)-\sum_{k=1}^{j}w_{ki}\left(n\right)y_{k}\left(n\right),j=1,\ldots ,p$$

and $z_{j}\left(n\right)={\left[z_{j1}\left(n\right),\ldots ,z_{jm}\left(n\right)\right]}^{T}$. The $z_{ji}\left(n\right)$ can be obtained from $z_{(j-1)i}\left(n\right)$ by:

(6) $$z_{ji}\left(n\right)=z_{(j-1)i}\left(n\right)-w_{ji}\left(n\right)y_{j}\left(n\right),j=2,\ldots ,p$$

**Figure 7:**
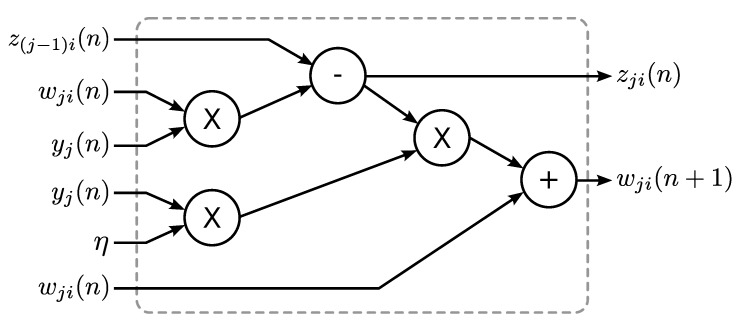
The hardware implementation of each module in the SWU unit of the GHA circuit for feature extraction.

**Figure 8:**
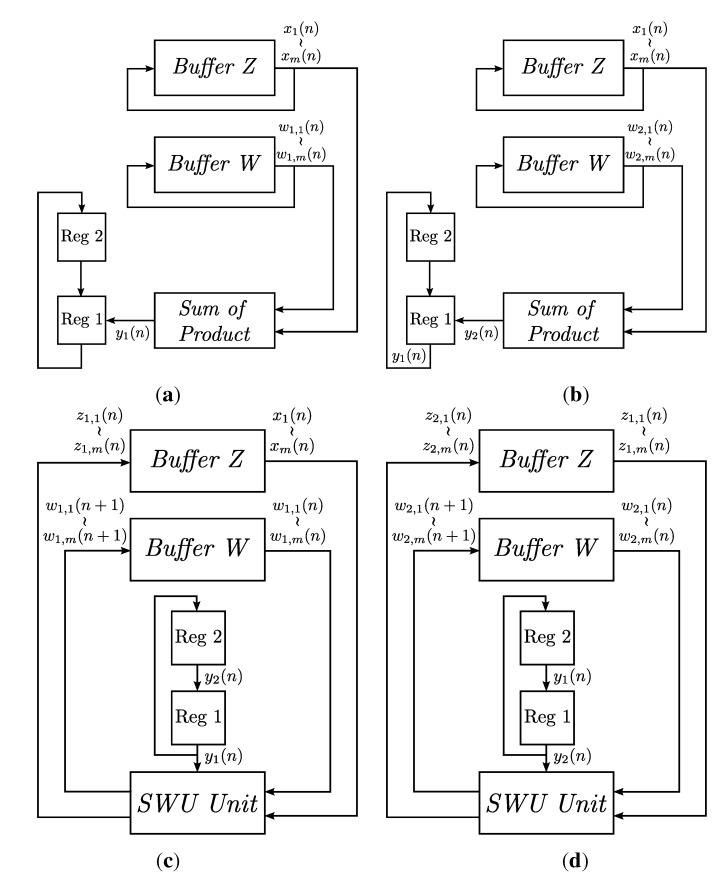
The operations of the basic single-channel GHA circuit for feature extraction for p=2: (**a**) the computation of $y_{1}\left(n\right)$ using the SOP circuit; (**b**) the computation of $y_{2}\left(n\right)$ using the SOP circuit; (**c**) the computation of $w_{1}\left(n+1\right)$ using the SWU unit; (**d**) the computation of $w_{2}\left(n+1\right)$ using the SWU unit.

When j=1, from Equations (5) and (6), it follows that $z_{0i}\left(n\right)=x_{i}\left(n\right)$. Therefore, the hardware implementation of Equations (4) and (6) is equivalent to that of Equation (3). Figure 7 depicts the hardware implementation of Equations (4) and (6). Because the dimensions of x(n), $w_{j}\left(n\right)$, $w_{j}\left(n+1\right)$ and $z_{j}\left(n\right)$ are *m*, replications of the circuit in Figure 7 may be desired to expedite the updating of weight vectors. The set of all of the replications of the circuit forms the SWU unit, where each individual replication is termed a module. Figure 8 shows the operations of the GHA circuit for p=2. Initially, x(n) is stored in Buffer Z. The weight vectors $w_{1}\left(n\right)$ and $w_{2}\left(n\right)$ are stored in Buffer W. As shown in Figure 8a, the SOP circuit first computes $y_{1}\left(n\right)$ based on x(n) and $w_{1}\left(n\right)$. It then finds $y_{2}\left(n\right)$ using x(n) and $w_{2}\left(n\right)$. Both $y_{1}\left(n\right)$ and $y_{2}\left(n\right)$ are stored in the two registers (denoted by Regs 1 and 2 in the figure) for subsequent synaptic weight updating operations in the SWU unit.

The operations of the SWU unit are shown in Figure 8c,d. In this example, there are *m* modules in the SWU unit. Buffer Z, Buffer W and two registers, which are used for SOP operations, also operate in conjunction with the SWU unit. Similar to the case for SOP operations, x(n) is stored in Buffer Z. The weight vectors $w_{1}\left(n\right)$ and $w_{2}\left(n\right)$ are stored in Buffer W. The results of the SOP operations, $y_{1}\left(n\right)$ and $y_{2}\left(n\right)$, are stored in the two registers (denoted by Regs 1 and 2 in the figure).

There are two steps for weight vector updating. At the first step, as shown in Figure 8c, the SWU unit computes the updated weight vector $w_{1}\left(n+1\right)$ based on x(n), $y_{1}\left(n\right)$ and $w_{1}\left(n\right)$. The $w_{1}\left(n+1\right)$ will be stored in Buffer W to replace $w_{1}\left(n\right)$. The SWU unit also provides $z_{1}\left(n\right)$, which will be stored in Buffer Z to replace x(n). At the second step, the SWU unit computes the updated weight vector $w_{2}\left(n+1\right)$ based on $z_{1}\left(n\right)$, $y_{2}\left(n\right)$ and $w_{2}\left(n\right)$. Figure 8d depicts the operations at the second step.

Note that the operations shown in Figure 8 are only for the simple cases, where all of the *m* elements of x(n), $w_{j}\left(n\right),z_{j}\left(n\right),j=1,2,$ can be used for the SOP and SWU circuits concurrently. When *m* is large, the area costs for implementing these circuits may be high. One way to solve this problem is to separate each of **x**, $w_{j}\left(n\right),z_{j}\left(n\right),j=1,2,$ into equal-sized segments. Buffer Z provides x(n) (or $z_{j}\left(n\right)$) one at a time. Similarly, the SOP and SWU circuits fetch $w_{j}\left(n\right)$ from Buffer W one segment at a time. Let *L* be the size of segments. It then follows that only *L* multipliers are required in the SOP circuit. Moreover, the SWU circuit contains only *L* modules. For large *m* and/or small *L*, the area costs may then be reduced at the expense of larger latency.

**Figure 9:**
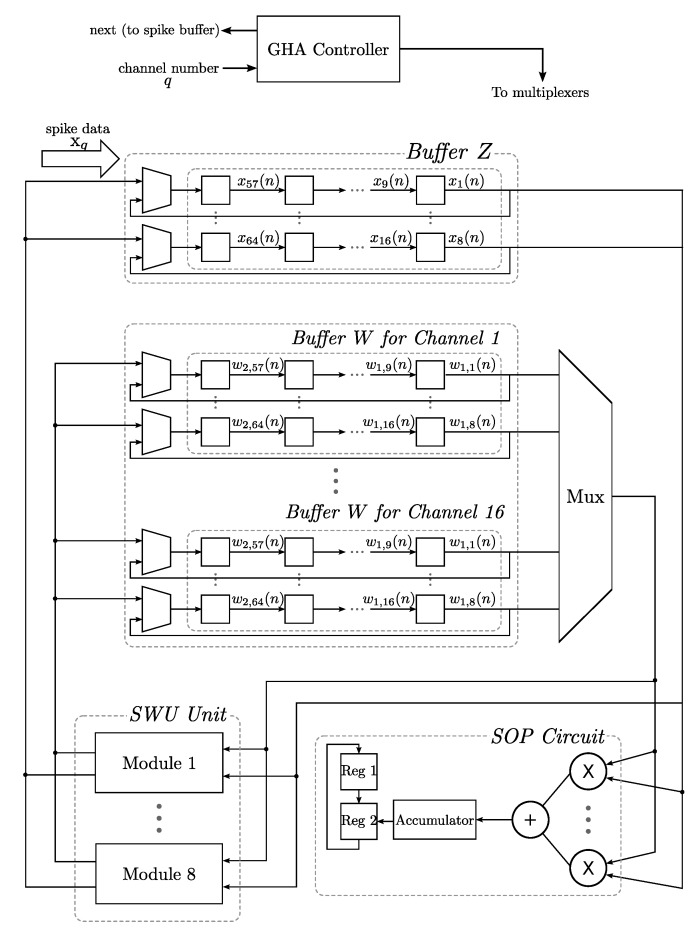
An example of the multi-channel GHA circuit for feature extraction with two principal components (p=2) and 16 channels (M=16). There are eight modules in the SWU unit and eight multipliers in the SOP circuit (L=8).

To extend the single-channel GHA circuit to a multi-channel one, the SOP circuit and the SWU circuit are shared by all channels for lowering the average area cost for each channel. Each channel q,q=1,...,M, needs to have its own Buffer W for storing its synaptic weight vectors. Figure 9 shows an example of the multi-channel GHA circuit for p=2 and M=16. That is, the circuit is able to support the GHA operations for two principal components and 16 channels. All of the channels share the same SOP circuit and SWU circuit. We set the length of segments L=8 so that there are eight multipliers in the SOP circuit and eight modules in the SWU circuit. In addition, because M=16, there are 16 Buffer W’s, with one for each channel. Each Buffer W is configured as a shift register supplying segments of the weight vectors one at a time. In this example, there are two weight vectors for each channel, and each weight vector is separated into *b* segments, where m=bL. Suppose that the dimension of spikes is 64 (*i.e*., m=64); each weight vector is separated into b=8 segments. Consequently, there are pb=16 stages in each Buffer W, where each stage holds the value of one segment. Moreover, since the multi-channel GHA circuit carries out the training one channel at a time, one Buffer Z is required in the circuit. The Buffer Z is also a shift register with b=8 stages, where each stage holds the value of one segment.

**Figure 10:**
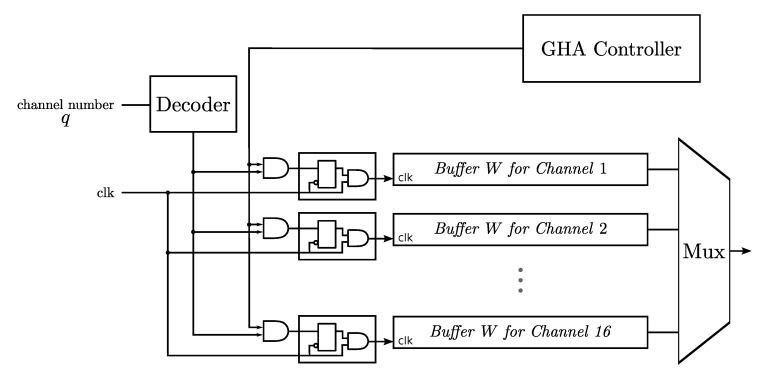
The clock gating circuit for lowering the dynamic power consumption of the multi-channel GHA circuit for feature extraction.

Similar to the cases for the NEO circuit and spike buffer, the CG technique is beneficial for lowering the power consumption of the multi-channel GHA circuit. This is because at most one Buffer W is used for training in the GHA circuit. Therefore, as shown in Figure 10, the CG circuit controls the supply of the system clock to the Buffer W of each channel. Only the channel selected for for GHA training obtains the system clock to activate its Buffer W.

### 2.5. Capacity Analysis of the Proposed Architecture

The maximum number of channels *M* supported by the proposed architecture is dependent on the sampling rate $r_{s}$ (with sampling period $T_{s}$) of the spikes, the clock rate $r_{c}$ (with sampling period $T_{c}$) of the circuit and the latency *P* of the proposed circuit. Recall that all of the detected spikes are stored in the spike buffer before they can be further processed by the GHA circuit. For any channel *h* in the circuit, a detected spike in that channel is said to be discarded for GHA training when the spike is over-written in the spike buffer by the next detected spike from the same channel *h* before it can be further processed by the GHA circuit. Given $r_{s}$, $r_{c}$ and *P*, the goal of this subsection is then to find the maximum number of channels *M* guaranteeing no discarded spikes in the spike buffer for any channel.

All of the detected spikes in the spike buffer are processed by the GHA circuit on a first-come-first-serve basis. For the sake of simplicity, assume that the memory unit for each channel in the spike buffer is able to hold only one spike. Under these conditions, the simplest scenario for a spike $x_{h}$ detected in a channel *h* is first considered. In the scenario, there is no detected spike for all of the remaining M-1 channels stored in the spike buffer. The GHA circuit is also in the idle state. In this case, the detected spike $x_{h}$ is immediately processed by the GHA circuit. Therefore, it will not be over-written by the next detected spike from channel *h*. Consequently, it will not be discarded by the proposed circuit for GHA training.

To find the maximum number of channels *M*, the worst case scenario is considered, where the detected spikes from all of the remaining M-1 channels are stored in the spike buffer and are not processed by the GHA circuit yet. In addition, the GHA circuit is currently busy. In this case, the circuit GHA needs to process *M* preceding spikes (*i.e*., the detected spikes stored in the spike buffer for all of the remaining M-1 channels and the detected spike currently processed by the GHA circuit) before it can process the $x_{h}$ for the channel *h* in the spike buffer. Because the latency for processing each detected spike is *P* clock cycles, it follows that the GHA circuit starts to process $x_{h}$ after MP clock cycles. Let *Q* be the minimum number of samples between the peak of successive spikes detected by the NEO circuit from the same channel. Assume that *Q* is the same for all of the channels. It then follows that $x_{h}$ is not overwritten and discarded when:

(7) $$MPT_{c}\leq QT_{s}$$

That is, the processing time by the GHA circuit for the preceding *M* detected spikes (*i.e*., $MPT_{c}$) should be less than the time interval between the peaks of two successive detected spikes from the same channel (*i.e*., $T_{s}$). Therefore, when the number of channels satisfies $M\leq \frac{QT_{s}}{PT_{c}}$, no detected spike will be discarded.

It is interesting to know that the NEO circuit imposes an additional limit on the number of channels *M*. It is desired that the NEO circuit is able to receive one sample from each channel in a single sampling period $T_{s}$. Based on the round robin scheme for fetching samples for different channels, as shown in Figure 3, it is therefore necessary that:

(8) $$MT_{c}\leq T_{s}$$

By comparing Equation (7) to Equation (8), it is concluded that the number of channels *M* needs to meet the condition in Equation (7) when Q<P. Otherwise, it should satisfy Equation (8). Therefore, the maximum number of channels, denoted by $M_{\max}$, satisfying Equations (7) and (8) is given by:

(9) $$M_{\max}=\left\{\begin{array}{cc} \left\lfloor \frac{Q}{P}\frac{T_{s}}{T_{c}}\right\rfloor & ifQ<P\\ \left\lfloor \frac{T_{s}}{T_{c}}\right\rfloor & ifQ\geq P \end{array}\right.$$

## 3. Experimental Results

In this section, the performance of the proposed architecture is evaluated. The area complexities of the proposed architecture are first analyzed. There are five types of area costs: the number of comparators, adders, multipliers, registers and multiplexers. All of the costs are expressed in terms of the asymptotic function (*i.e*., the Big*O* function) in the tables. Table 1 shows the area complexities of the GHA circuit. It can be observed from Table 1 that the numbers of adders and the multipliers of the GHA are independent of the number of channel *M*. This is because all of the channels share the same computation core (*i.e*., the SWU and SOP circuits) for the GHA training. The computation core of the GHA circuit is able to process the detected spikes one segment at a time. Therefore, the numbers of adders and multipliers grow with the dimension of the segments *L*. On the other hand, because the circuit needs to store the synaptic weight vectors for each and every channels, the number of registers is dependent on the number of channels *M*, the number of weight vectors for each channel *p* and the dimension of each vector *m*.

Based on the analytical results in Table 1, the overall area complexities of the proposed spike sorting circuit are summarized in Table 2. The area complexities of the NEO circuit and spike buffer are also included in the table for evaluation. We can observe from Table 2 that the number of registers in both the NEO circuit and spike buffer are also dependent on the number of channels *M*. This is because it is necessary to store spike trains from all of the *M* channels for detection. Moreover, each channel needs to have its own memory unit to hold the detected spikes for the subsequent GHA training. Therefore, the number of registers in the NEO circuit, spike buffer and GHA circuit all increase with the number of channels *M*. By contrast, because the NEO circuit has only a fixed number of adders and multipliers independent of *M*, the overall area costs for the adders and multipliers in the spike sorting circuit are dependent only on *L*.

**Table 1:** The area complexities of the GHA circuit.

	Comparators	Adders	Multipliers	Registers	Multiplexers
SWU Circuit	0	O(L)	O(L)	0	0
SOP Circuit	0	O(L)	O(L)	O(1)	0
Buffer Z	0	0	0	O(m)	O(L)
Buffer W	0	0	0	O(Mpm)	O(ML)
GHA Circuit	0	O(L)	O(L)	O(Mpm)	O(ML)

**Table 2:** The area complexities of the proposed spike sorting circuit.

	Comparators	Adders	Multipliers	Registers	Multiplexers
NEO Circuit	O(1)	O(1)	O(1)	O(Mm)	0
Spike Buffer	0	0	0	O(Mm)	O(1)
GHA Circuit	0	O(L)	O(L)	O(Mpm)	O(ML)
Total	O(1)	O(L)	O(L)	O(Mpm)	O(ML)

We next evaluate the hardware resource consumption of the ASIC implementation of the proposed circuit with clock gating. The implementation is based on the TSMC90-nm technology. The gate level design platform is Synopsys Design Compiler. Table 3 shows the area (μm^2^) of the proposed circuit for different numbers of channels *M* and segment lengths *L*. For all of the experiments shown after Table 3, the dimension of spikes and weight vectors is m=64. The number of principal components is p=2. From Table 3, we see that the area of the proposed circuit grows with *M* and *L*, which is consistent with the results shown in Table 2. Table 4 shows the normalized area (μm^2^ per channel) of the proposed architecture. The normalized area is defined as the area of the circuit divided by the number of channels *M*. The normalized area can be viewed as the average area cost per channel. For a fixed *L*, it can be observed from the table that the normalized area decreases as the number of channels *M* increases. In particular, when L=8, the normalized area decreases from 162,255 μm^2^/ch. for M=2 to 80,503 μm^2^/ch. for M=64. The reduction in normalized area for large *M* is due to the fact that all of the channels share the same computation cores in the NEO circuit (*i.e*., the NEO detection unit) and GHA circuit (*i.e*., the SWU unit and the SOP circuit). The area of computation cores is independent of *M*. Therefore, the average area per channel decreases as *M* increases. Consequently, because of the hardware resource sharing scheme, the proposed architecture shows a higher efficiency in area costs for a larger number of channels.

**Table 3:** The area (μm^2^) of the proposed circuit for different numbers of channels *M* and segment lengths *L*.

Segment	Number of Channels *M*					
Length *L*	2	4	8	16	32	64
2	241,090	395,565	701,479	1,318,032	2,548,962	5,010,148
4	268,776	423,957	731,167	1,350,163	2,586,267	5,057,620
8	324,509	480,852	790,654	1,414,494	2,661,096	5,152,185
16	435,478	594,419	909,403	1,543,228	2,810,642	5,341,566
32	658,196	822,560	1,148,565	1,802,752	3,114,285	5,728,802

**Table 4:** The normalized area (μm^2^/ch.) of the proposed circuit for different numbers of channels *M* and segment lengths *L*.

Segment	Number of Channels *M*					
Length *L*	2	4	8	16	32	64
2	120,545	98,891	87,685	82,377	79,655	78,284
4	134,388	105,989	91,396	84,385	80,821	79,025
8	162,255	120,213	98,832	88,406	83,159	80,503
16	217,739	148,604	113,675	96,452	87,833	83,462
32	329,098	205,640	143,571	112,672	97,321	89,513

Although a larger segment length *L* increases the area of the proposed architecture, the latency *P* of the GHA circuit is reduced. As a result, the maximum number of channels $M_{\max}$ is increased given fixed sampling period $T_{s}$ and clock period $T_{c}$. Table 5 shows the latency *P* (in clock cycles) of the GHA circuit for various *L* values. The maximum number of channels $M_{\max}$ for each *L* value is also shown. The $M_{\max}$ is computed by Equation (9). The sampling rate of spike trains is set to be $r_{s}=24,000$ samples/s. There are three clock rates considered in the table: $r_{c}$ = 0.5 MHz, 1 MHz and 2 MHz. The minimum number of samples between the peak of successive spikes detected by the NEO circuit from the same channel is Q=16. We can observe from Table 5 that the latency *P* is lowered to 16 clock cycles when *L* is 32. Because *Q* is 16, from Equation (9), it can be concluded that the maximum number of channels of the proposed architecture is identical to that limited by the NEO circuit (*i.e*., $\left\lfloor \frac{T_{s}}{T_{c}}\right\rfloor$) when *L* is 32.

When a larger *Q* is allowed, the maximum number of channels $M_{\max}$ supported by the proposed architecture may be increased. Table 6 shows the $M_{\max}$ of the proposed algorithm for Q=32. By comparing Table 5 and Table 6, it can be observed that $M_{\max}$ may increases two-fold for the circuits when *Q* increases from 16 to 32. In particular, when L=8 and $r_{s}=1$ MHz, the $M_{\max}$ is 16 and 33 for Q=16 and 32, respectively. These facts can be further elaborated in Table 7 for various combinations of *Q*, *L* and $r_{s}$. When L=4, we can see from Table 7 that $M_{\max}$ grows linearly with *Q* for all of the clock rates $r_{c}$. The $M_{\max}$ for L=8 is larger than that for L=4 given the same *Q* and $r_{s}$. In addition, it also grows with *Q* for small *Q* values. Moreover, since the latency for L=8 is P=40, the proposed architecture achieves the maximum number of channels limited by the NEO circuit when *Q* is larger or equal to 40. By contrast, the proposed architecture with L=4 supports the maximum channel capacity when *Q* reaches 72. Therefore, when both smaller *Q* values and a larger number of channels are desirable for GHA training, larger *L* values may be preferred. Otherwise, a smaller *L* value could be selected for the spike sorting due to lower area costs.

**Table 5:** The latency *P* of the GHA circuit and the maximum number of channels $M_{\max}$ for various segment lengths *L* and clock rates $r_{c}$ of the proposed spike sorting circuit with clock gating. The sampling rate of spike trains is $r_{s}=$ 24,000 samples/s. The minimum number of samples between the peak of successive spikes from the same channel is assumed to be Q=16.

Segment	Latency	Max. No. of Channels $M_{\max}$		
Length *L*	*P*	$r_{c}$ = 0.5 MHz	$r_{c}$ = 1 MHz	$r_{c}$ = 2 MHz
		$\lfloor \frac{T_{s}}{T_{c}}\rfloor =20$	$\lfloor \frac{T_{s}}{T_{c}}\rfloor =41$	$\lfloor \frac{T_{s}}{T_{c}}\rfloor =83$
1	264	1	2	5
2	136	2	4	9
4	72	4	9	18
8	40	8	16	33
16	24	13	27	55
32	16	20	41	83

**Table 6:** The latency *P* of the GHA circuit and the maximum number of channels $M_{\max}$ for various segment lengths *L* and clock rates $r_{c}$ of the proposed spike sorting circuit. The sampling rate of spike trains is $r_{s}=$ 24,000 samples/s. The minimum number of samples between the peak of successive spikes from the same channel is assumed to be Q=32.

Segment	Latency	Max. No. of Channels $M_{\max}$		
Length *L*	*P*	$r_{c}$ = 0.5 MHz	$r_{c}$ = 1 MHz	$r_{c}$ = 2 MHz
		$\lfloor \frac{T_{s}}{T_{c}}\rfloor =20$	$\lfloor \frac{T_{s}}{T_{c}}\rfloor =41$	$\lfloor \frac{T_{s}}{T_{c}}\rfloor =83$
1	264	2	5	10
2	136	4	9	19
4	72	9	18	37
8	40	16	33	66
16	24	20	41	83
32	16	20	41	83

**Table 7:** The maximum number of channels $M_{\max}$ for various combinations of *Q*, *L* and $r_{c}$.

		Max. No. of Channels $M_{\max}$		
		$r_{c}$ = 0.5 MHz	$r_{c}$ = 1 MHz	$r_{c}$ = 2 MHz
		$\lfloor \frac{T_{s}}{T_{c}}\rfloor =20$	$\lfloor \frac{T_{s}}{T_{c}}\rfloor =41$	$\lfloor \frac{T_{s}}{T_{c}}\rfloor =83$
Q=16	L=4	4	9	18
	L=8	8	16	33
Q=32	L=4	9	18	37
	L=8	16	33	66
Q=48	L=4	13	27	55
	L=8	20	41	83
Q=64	L=4	18	37	74
	L=8	20	41	83

Table 8 shows the power dissipation of the proposed architecture for L=8 with different clock rates $r_{c}$. When $r_{c}=1$ MHz, the numbers of channels implemented are M=8, 16 and 32. The circuit with M=64 is not implemented because the maximum number of channels $M_{\max}$ is only 41, even for large *Q*, such as Q=64, as shown in Table 7. When $r_{c}=2$ MHz, we have implemented the circuit for M=8, 16, 32 and 64. For each *M* value considered in Table 8, its normalized power consumptions with and without clock gating are measured. The percentage of power reduction from the circuit without clock gating to the circuit with clock gating for each *M* is also included. The power consumption measurement is performed numerically by Synopsys Prime Time.

**Table 8:** The performance of the proposed spike sorting circuit for L=8.

Number of	Clock	Normalized Power		Power
Channels *M*	Rates $r_{c}$	No Clock Gating	Clock Gating	Reduction
8	1 MHz	156.1 μW/ch.	114.3 μW/ch.	26.78 %
16	1 MHz	133.1 μW/ch.	91.4 μW/ch.	31.33 %
32	1 MHz	120.5 μW/ch.	78.7 μW/ch.	34.69 %
8	2 MHz	215.4 μW/ch.	152.4 μW/ch.	29.2 %
16	2 MHz	179.1 μW/ch.	115.5 μW/ch.	35.5 %
32	2 MHz	159.3 μW/ch.	95.3 μW/ch.	40.18 %
64	2 MHz	150.0 μW/ch.	85.8 μW/ch.	42.80 %

It can be observed from Table 8 that the clock gating technique is able to reduce the power consumption of the proposed circuit. The reduction is due to the lower dynamic power consumption by disabling clock supply to the buffers of the channels currently not engaged in GHA training. We can also see from Table 8 that, as the number of channels and/or the clock rate increase, the reduction in power consumption increases. In particular, for M=64 and $r_{c}=2$ MHz, the power consumption is lowered from 150.0 μW/ch. without clock gating to 85.8 *μ*W/ch. with clock gating. The reduction is therefore 42.80%. For a lower number of channels and/or lower clock rate, the power reduction is still above 26.78%. The clock gating technique is therefore beneficial for the low power design of the spike sorting system.

To further demonstrate the effectiveness of the proposed architecture, Table 9 compares the proposed architecture with the existing ASIC implementations for spike sorting. Direct comparisons among these architectures may be difficult because these architectures are implemented by different technologies and are based on different spike detection and/or feature extraction algorithms. Moreover, their operation frequencies are different. Nevertheless, it can be observed from Table 9 that, as compared with the existing architectures, the proposed architecture provides effective area-power performance while supporting both spike detection and feature extraction functions. In particular, subject to the same technology (*i.e*., 90 nm), clock rate (1 MHz) and spike dimension (*i.e*., 64), the proposed architecture has lower power dissipation (78.819 μW/ch. *vs*. 521 μW/ch.) and lower area (83,159 μm^2^/ch. *vs*. 255,495 μm^2^/ch.) as compared to the architecture in [6]. The proposed architecture has superior performance because it adopts the GHA training operations for feature extraction. Therefore, there is no need to incorporate memory blocks for covariance matrices of the training data, which are required by [6]. A variant of PCA, termed Streaming Pattern dIscoveRy in multIple Time-series (SPIRIT), has been implemented by ASIC in [21] without the employment of memory blocks for covariance matrices. Although the SPIRIT circuit is able to consume lower average power, it has a higher area cost as compared to the proposed circuit. In addition, the SPIRIT circuit supports only one channel without spike detection and alignment capabilities. The proposed circuit therefore provides a superior solution when a low-cost implementation of multi-channel spike detection and feature extraction is desired.

**Table 9:** Comparison between existing architectures and that proposed. SPIRIT, streaming pattern discovery in multiple time-series.

**Architecture**	[6]	[7]	[12]	[21]	Proposed	Proposed
**Normalized**	255,495	1,770,000	116,000	268,000	83,159	80,503
**Area**	μm^2^/ch.	μm^2^/ch.	μm^2^/ch.	μm^2^/ch.	μm^2^/ch.	μm^2^/ch.
**Normalized**	521	256.875	95.6	8.589	78.719	85.828
**Power**	μW/ch.	μW/ch.	μW/ch.	μW/ch.	μW/ch.	μW/ch.
**Clock Rate**	1 MHz	N/A	16 MHz	281.25 KHz	1 MHz	2 MHz
**# of Channels**	1	16	16	1	32	64
**Spike Dimension**	64	N/A	64	64	64	64
**Technology**	90 nm	350 nm	180 nm	130 nm	90 nm	90 nm
**Detection**	No	NEO	Absolute	No	NEO	NEO
**Feature Extraction**	PCA	PCA	No	SPIRIT	GHA	GHA

**Figure 11:**
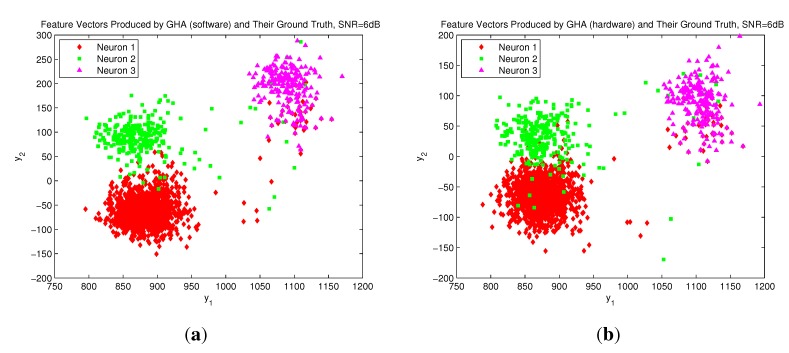
The distribution of the feature vectors extracted by GHA hardware/software implementations for spike trains generated by three neurons with SNR = 6 dB: (**a**) feature vectors produced by the GHA software; (**b**) feature vectors produced by the GHA circuit.

In addition to being effective for hardware implementation, the GHA architecture has comparable accuracy for feature extraction as compared to its software counterpart and the basic PCA algorithm. The software versions of GHA and PCA algorithms are implemented by MATLAB with double precision floating numbers. The GHA circuit is based on 17-bit fixed point numbers. The simulator developed in [22] is adopted to generate extracellular recordings. The simulation gives access to ground truth about spiking activity in the recording and, thereby, facilitates a quantitative assessment, since the features of the spike trains are known *a priori*. Table 10 shows the classification success rates (CSRs) of the fuzzy C-means (FCM) algorithm [23,24] by clustering the feature vectors produced by the GHA and PCA hardware/software implementations for two and three neurons, respectively. The CSR is defined as the number of spikes that are correctly classified divided by the total number of spikes. Spike trains with different SNR levels are considered in the table. It can be observed in Table 10 that the CSRs of the FCM algorithm for the data produced by GHA and PCA hardware/software implementations given the same SNR level are comparable. The GHA circuit has slight degradation in CSR, because the precision of its number representation is finite (*i.e*., 17 bits). To further reveal the effectiveness of the GHA circuit, Figure 11 shows the distribution of the feature vectors extracted by the GHA circuit and the GHA software MATLAB codes for spike trains generated by three neurons with SNR = 6 dB. From Figure 11, it can be observed that the GHA hardware and software implementations have a similar distribution of feature vectors. Therefore, they may produce comparable CSRs, as revealed in Table 10. The GHA circuit therefore is an effective alternative for hardware implementation when the consumption of hardware resource and power and the classification performance are the important concerns. All of these facts show the effectiveness of the proposed architecture.

**Table 10:** The classification success rates (CSRs) of the feature vectors produced by the GHA and PCA hardware/software implementations for two and three neurons.

**Number of Neurons**	2			3		
**SNR (dB)**	6	8	10	6	8	10
**PCA Software**	99.51%	99.64%	99.39%	95.93%	96.91%	97.41%
**GHA Software**	99.39%	99.52%	99.33%	95.50%	96.53%	97.19%
**GHA Hardware**	97.49%	97.92%	97.31%	92.24%	94.80%	95.95%

## 4. Conclusions

The proposed architecture has been implemented by ASIC with TSMC 90-nm technology for hardware performance evaluation. Several design examples supporting up to 64 channels and operating up to 2 MHz clock rates are evaluated. The proposed architecture employs the computation core sharing and clock gating techniques for enhancing the hardware performance. Experimental results show that the computation core sharing and clock gating are able to reduce the average area cost and power consumption per channel, respectively. In particular, when the SWU unit of the GHA circuit contains eight modules, the normalized area decreases by 50.38% from 162,255 μm^2^/ch. for two channels to 80,503 μm^2^/ch. for 64 channels. Moreover, the normalized power consumption for 64 channels operating at 2 MHz clock rate reduces by 42.80% from 150.0 μW/ch. without clock gating to 85.8 μW/ch with clock gating. In addition to having efficient area and power performance, the proposed architecture offers comparable classification accuracy to that of the software implementations of GHA and PCA algorithms. In fact, for the case of two neurons, the proposed architecture attains CSRs above 97%. The proposed architecture therefore provides an effective solution to the applications where the implantable spike sorting circuits with low power consumption, low area costs and high accuracy spike sorting are desired.
